# Investigation of *FADS* Gene Cluster Single Nucleotide Polymorphisms in End-Stage Renal Disease Compared With Normal Controls

**DOI:** 10.3389/fgene.2021.716151

**Published:** 2021-09-16

**Authors:** Amirhossein Miladipour, Mahdi Gholipour, Kasra Honarmand Tamizkar, Atefe Abak, Vahid Kholghi Oskooei, Mohammad Taheri, Soudeh Ghafouri-Fard

**Affiliations:** ^1^Urology and Nephrology Research Center, Shahid Beheshti University of Medical Sciences, Tehran, Iran; ^2^Men’s Health and Reproductive Health Research Center, Shahid Beheshti University of Medical Sciences, Tehran, Iran; ^3^Phytochemistry Research Center, Shahid Beheshti University of Medical Sciences, Tehran, Iran; ^4^Department of Laboratory Sciences, School of Paramedical Sciences, Torbat Heydariyeh University of Medical Sciences, Torbat Heydariyeh, Iran; ^5^Neuroscience Research Center, Torbat Heydariyeh University of Medical Sciences, Torbat Heydariyeh, Iran; ^6^Skull Base Research Center, Loghman Hakim Hospital, Shahid Beheshti University of Medical Sciences, Tehran, Iran; ^7^Department of Medical Genetics, School of Medicine, Shahid Beheshti University of Medical Sciences, Tehran, Iran

**Keywords:** *FADS1*, *FADS2*, *FADS3*, polymorphisms, hemodialysis

## Abstract

End-stage renal disease (ESRD) is a public health problem with a high burden. The condition is associated with abnormalities in lipid metabolism. The fatty acid desaturase (FADS) gene cluster includes three genes that are significantly correlated with a number of pathologic conditions related to abnormal lipid levels. In the current study, we genotyped rs174556, rs99780, and rs7115739 single nucleotide polymorphisms within the *FADS* cluster in a population of ESRD patients and healthy controls. The rs174556 of the *FADS1* gene and rs99780 of the *FADS2* gene were not associated with the risk of ESRD in any inheritance model. However, the rs7115739 of *FADS3* was associated with the risk of ESRD in all models except for the recessive model. The T allele of this SNP was significantly less prevalent among cases compared with controls [odds ratio (OR) (95% CI) = 0.44 (0.25–0.77), *P* value = 0.004]. GT and TT genotypes has been shown to decrease the risk of ESRD in a codominant model [OR (95% CI) = 0.49 (0.26–0.92) and OR (95% CI) = 0.18 (0.02–1.6), respectively; *P* value = 0.019]. In the dominant model, GT + TT status was associated with lower risk of ESRD [OR (95% CI) = 0.45 (0.24–0.82), *P* value = 0.0078]. Assessment of association between this SNP and risk of ESRD in an overdominant model revealed that GT genotype decreases the risk of this condition [OR (95% CI) = 0.5 (0.27–0.94), *P* value = 0.029]. Taken together, the rs7115739 of *FADS3* is suggested as a putative modulator of the risk of ESRD in the Iranian population.

## Introduction

End-stage renal disease (ESRD) is a public health problem all over the world with an increasing trend in its incidence in both developed and developing countries (Haghighi et al., 2002; Renal Du System (USRDS), 2010; Mousavi et al., 2014). The prevalence of chronic renal disease is estimated to be around 13.4% throughout the world (Lv and Zhang, 2019). Notably, between 4.902 and 7.083 million individuals worldwide have ESRD and are in need of kidney transplantation (Lv and Zhang, 2019). The total rise in the prevalence of ESRD is mostly due to the rise in the prevalence of a number of associated risk factors including diabetes mellitus and hypertension (Lv and Zhang, 2019). Due to the high burden of this disease and the unavailability of renal replacement therapy in some regions (Lv and Zhang, 2019), it is necessary to find the underlying causes and genetic risk factors for the development of ESRD.

Previous studies have shown substantial elevation of the risk of ESRD in patients with a family history of this condition (Satko et al., 2005), implying the presence of genetic risk factors for ESRD. Genome-wide association studies (GWAS) have led to the identification of more than 100 loci for chronic kidney disease and its related conditions (Pattaro et al., 2016; Wuttke and Köttgen, 2016; Gorski et al., 2017). A number of newly identified SNPs have been shown to be associated with transcript levels of *SYPL2*, *SDCCAG8*, *MANBA*, *KBTBD2*, *PTPRO*, and *SPATA33* genes, thus providing evidence that these genes are potential candidates for the observed associations (Pattaro et al., 2016). Moreover, a number of these loci, including *SDCCAG8*, *LRP2*, *IGFBP5*, *SKIL*, *UNCX*, *KBTBD2*, *A1CF*, *KCNQ1*, AP5B1, *PTPRO*, *TP53INP2*, and *BCAS1* have been shown to be consistently associated with this trait based on random-effect meta-analyses (Pattaro et al., 2016).

The fatty acid desaturase (FADS) gene cluster includes three genes that are significantly correlated with a number of pathologic conditions such as abnormal lipid levels, fatty liver disorder, and diabetes mellitus (Gromovsky et al., 2018). Genetic polymorphisms within the *FADS1* and *FADS2* genes have been associated with fatty acid metabolic pathways via changing methylation patterns and the expression of genes (He et al., 2018). Moreover, these polymorphisms have been found to affect the plasma levels of fatty acids and desaturase in diabetic patients suffering from coronary artery disease (Li et al., 2016).

End-stage renal disease has been shown to be associated with the premature development of atherosclerosis and mortality from coronary artery diseases due to increased levels of oxidative stress, as well as abnormalities in lipid metabolism (Vaziri, 2009).

Based on the association between ESRD and abnormal lipid metabolism, genes participating in the lipid metabolism might be associated with the development of ESRD. To test this hypothesis, we designed the current study to appraise the association between rs174556, rs99780, and rs7115739 single nucleotide polymorphisms (SNPs) within the *FADS* cluster on 11q12–q13.1 and the risk of ESRD in 164 Iranian patients admitted to Labbafinejad Hospital, Tehran, Iran compared with 171 age- and sex-matched controls.

These SNPs have been selected based on the results of previous studies regarding their role in a number of metabolism-related diseases. The *FADS1* rs174556 has been suggested to be associated with acute coronary syndrome in a population of Chinese patients, particularly those with hypertension (Song et al., 2013). Among several SNPs within the *FADS* cluster, rs174556 has been found to be representative of the disease-associated block in a study (Campoy et al., 2021). rs99780 has been among SNPs associated with alteration of plasma levels of (n-6) and (n-3) essential fatty acids and erythrocyte phospholipids (Xie and Innis, 2008). Finally, rs7115739 has been shown to be associated with total cholesterol levels among Chinese patients (Wu et al., 2017).

## Materials and Methods

### Patients and Controls

The current study included a total of 164 cases (75 women and 89 men) and 171 controls (81 women and 90 men). Table 1 shows the description of cases and controls.

**TABLE 1:** Demographic data of cases and controls.

**Variables**	**Cases**	**Controls**
Female/Male [no. (%)]	75 (45%)/89 (55%)	81 (47%)/90 (53%)
Age (mean ± SD, Y)	56.2 ± 2.7	52.3 ± 2.4
Age range (Y)	17–85	22–79

All cases had severe ESRD. Cases were recruited from the hemodialysis Ward of Labbafinejad Hospital, Tehran, Iran during 2019–2020. Controls were healthy subjects with no personal or familial history of chronic renal disorder. Exclusion criteria were malignancies or autoimmune disorders. Patients with other concurrent disorders were also excluded from the study. The study protocol was approved by the Ethical Committee of Shahid Beheshti University of Medical Sciences (IR. SBMU.UNRC.1398.10). All cases and controls signed the informed consent forms.

### Genotyping

Genomic DNA was isolated from venous blood specimens of all recruited persons using the standard salting-out method (Chacon-Cortes and Griffiths, 2014). The tetra-primer ARMS-PCR method (Medrano and de Oliveira, 2014) was used for the genotyping of rs174556, rs99780, and rs7115739 SNPs. For this purpose, 75 ng of genomic DNA, 0.30 pmol of each outer primer, 0.5 pmol of the forward inner primer, 0.80 pmol of the reverse inner primer, and Taq DNA polymerase master mix red (Ampliqon, Denmark). The PCR condition consisted of a preliminary denaturation at 94°C for 5 min, 35 cycles of 95°C for 45 s, specific annealing temperatures for 40 s, and 72°C for 60 s, with the final extension of 72°C for 5 min. The rs174556 (NC_000011.9:g.61580635C > T) is an intron variant in the *FADS1* gene close to rs981340263 and rs2066943471. The rs99780 (NC_000011.9:g.61596633C > T) is also an intron variant in *FADS2* near rs1299703736. Finally, rs7115739 (NC_000011.9:g.61641717T > G) is an intron variant of *FADS3*^1^. Primer sequences and PCR conditions for genotyping experiments are shown in Table 2.

**TABLE 2:** Primer sequences and PCR conditions for genotyping experiments.

**Genetic polymorphism**	**Primer sequence**	**Tm**	**Annealing temperature**	**PCR product size (bp)**
*FADS1* (rs174556)	Forward inner primer (C allele):	62.5°C	57°C	189 (C allele)
	5′- ACTGACTGTGATTACTATGACTGTGCTC-3′			
	Reverse inner primer (T allele):	62.5°C		268 (T allele)
	5′-ATGGGTGGAAGGAGAGTCTAGATTGA-3′			
	Forward outer:	62.5°C		404 (two outer primers)
	5′-TGAGCCTGTTATCTCATCTGTAAATGTGT-3′			
	Reverse outer:	62.5°C		
	5′-GGGCCTTGTGAAGAAGTATATGAACTCT-3′			
*FADS2* (rs99780)	Forward inner primer (T allele):	65°C	59°C	218 (T allele)
	5′-AATATTAACATGGGAAAAACCGCCCCGT-3′			
	Reverse inner primer (C allele):	66°C		297 (C allele)
	5′-AATATATGGAGCGTGGGAACCCGAGATG-3′			
	Forward outer:	65°C		460 (two outer primers)
	5′-TAATATAGGTTTGTCTGGAGGCAGGGACTC-3′			
	Reverse outer:	64.5°C		
	5′-ATATTATAGACGGACCTGTTGCCAAGCC-3′			
*FADS3* (rs7115739)	Forward inner (T allele):	63°C	61°C	152 (T allele)
	5′-TATACCCGATCTTGGGCCTGATATTAGT-3′			
	Reverse inner (G allele):	63.5°C		260 (G allele)
	5′-TATTATTGACATTTCTGGCTCCACCGC-3′			
	Forward outer:	64°C		358 (two outer primers)
	5′-TAATGTGGGTTGGGAGAACAGCTGAA-3′			
	Reverse outer:	63.5°C		
	5′-ATGTACACTTCTCCTTCATGATCTCTCCC-3′			

### Statistical Methods

Genotype and allele frequencies of rs174556, rs99780, and rs7115739 were compared between cases and controls in allelic, dominant, recessive, codominant, overdominant, and log-additive models (Horita and Kaneko, 2015) through calculation of odds ratio (OR) and 95% confidence intervals (CI). *P* < 0.05 was considered as significant. For each SNP, a major allele (M) and a minor allele (m) have been considered. Therefore, genotypes consisted include major allele homozygote (MM), heterozygote (Mm), and minor allele homozygote (mm). In the dominant model, comparison was made between MM and Mm + mm. In the recessive model, MM + Mm were compared with mm. In the overdominant model, it is assumed that the heterozygote has the most robust effect, thus it compares MM + mm with Mm. Codominant models such as additive and log-additive models assume that risk is increased/decreased in a stepwise manner from MM to Mm and to mm (Horita and Kaneko, 2015).

## Results

The current study included a total of 164 cases (75 women and 89 men) and 171 controls (81 women and 90 men). Table 3 shows the clinical variables assessed in cases and controls. Cases and controls were matched in all variables except for albumin levels, which were significantly lower among cases compared with controls, which may well be due to impaired renal function. However, such difference in albumin levels cannot affect genotypes. Thus, it was not necessary to match cases and controls in this parameter.

**TABLE 3:** Clinical variables assessed in cases and controls.

**Variables**	**Cases**	**Controls**
TC (mean ± SD, mg/dL)	168.425.2	172.421.2
HDL (mean SD, mg/dL	42.561.79	39.241.6
LDL (mean SD, mg/dL)	108.273.65	98.263.59
TG (mean SD, mg/dL)	154.7119.8	159.6719.2
Albumin (mean SD, g/dL)	3.721.2	5.211.6
BMI (mean SD, kg/m^2^)	23.11.5	23.51.8

Genetic associations were assessed in different inheritance models (Horita and Kaneko, 2015). For rs174556, 47, 110, and 14 controls subjects had CC, CT, and TT genotypes, respectively. Among cases, these figures were 48, 107, and 9, respectively. For rs99780, 85, 69, and 17 control subjects were found to have CC, CT, and TT genotypes, respectively. A total of 88, 64, and 12 patients had CC, CT, and TT genotypes of rs99780, respectively. Finally, 134, 32, and 5 controls and 146, 17, and 1 patient(s) had GG, GT, and TT genotypes, respectively.

The rs174556 of *FADS1* gene and rs99780 of *FADS2* gene were not associated with risk of ESRD in any inheritance model. However, the rs7115739 of *FADS3* was associated with risk of ESRD in all models except for recessive model. T allele of this SNP was significantly less prevalent among cases compared with controls [OR (95% CI) = 0.44 (0.25–0.77), *P* value = 0.004]. GT and TT genotypes have been identified as protective genotypes against ESRD in the codominant model [OR (95% CI) = 0.49 (0.26–0.92) and OR (95% CI) = 0.18 (0.02–1.6), respectively; *P* value = 0.019]. In the dominant model, GT + TT status was associated with lower risk of ESRD [OR (95% CI) = 0.45 (0.24–0.82), *P* value = 0.0078]. Assessment of association between this SNP and risk of ESRD in overdominant model revealed that GT genotype decreases risk of this condition [OR (95% CI) = 0.5 (0.27–0.94), *P* value = 0.029]. Table 4 shows *FADS1*–*3* polymorphisms and ESRD.

**TABLE 4:** Results of association between *FADS1*–*3* polymorphisms and ESRD.

**Gene**	**Locus**	**Model**	**Genotype**	**Controls**	**Patients**	**Odds ratio**	***P* value**
*FADS1*	rs174556	Allele	C	204(59.6%)	203(61.9%)	1	0.55
			T	138(40.4%)	125(38.1%)	0.91 (0.67–1.24)	
		Codominant	CC	47(27.5%)	48(29.3%)	1.00	0.61
			CT	110(64.3%)	107(65.2%)	0.95 (0.59–1.54)	
			TT	14(8.2%)	9(5.5%)	0.63 (0.25–1.59)	
		Dominant	CC	47(27.5%)	48(29.3%)	1.00	0.71
			CT + TT	124(72.5%)	116(70.7%)	0.92 (0.57–1.47)	
		Recessive	CC + CT	157(91.8%)	155(94.5%)	1.00	0.33
			TT	14(8.2%)	9(5.5%)	0.65 (0.27–1.55)	
		Overdominant	CC + TT	61(35.7%)	57(34.8%)	1.00	0.86
			CT	110(64.3%)	107(65.2%)	1.04 (0.66–1.63)	
		Log-additive				086 (0.59–1.27)	0.46
*FADS2*	rs99780	Allele	C	239(69.9%)	240(73.2%)	1	0.35
			T	103(30.1%)	88(26.8%)	0.85 (0.61–1.19)	
		Codominant	CC	85(49.7%)	88(53.7%)	1.00	0.62
			CT	69(40.4%)	64(39%)	0.90 (0.59–1.54)	
			TT	17(9.9%)	12(7.3%)	0.68 (0.31–1.51)	
		Dominant	CC	85(49.7%)	88(53.7%)	1.00	0.47
			CT + TT	86(50.3%)	76(46.3%)	0.85 (0.56–1.31)	
		Recessive	CC + CT	154(90.1%)	152(92.7%)	1.00	0.39
			TT	17(9.9%)	12(7.3%)	0.72 (0.33–1.55)	
		Overdominant	CC + TT	102(59.6%)	100(61%)	1.00	0.8
			CT	69(40.4%)	64(39%)	0.95 (0.61–1.47)	
		Log-additive				0.85 (0.61–1.19)	0.35
*FADS3*	rs7115739	Allele	G	300(87.7%)	309(94.2%)	1	0.004
			T	42(12.3%)	19(5.8%)	0.44 (0.25–0.77)	
		Codominant	GG	134(78.4%)	146(89%)	1.00	0.019
			GT	32(18.7%)	17(10.4%)	0.49 (0.26–0.92)	
			TT	5(2.9%)	1(0.6%)	0.18 (0.02–1.6)	
		Dominant	GG	134(78.4%)	146(89%)	1.00	0.0078
			GT + TT	37(21.6%)	18(11%)	0.45 (0.24–0.82)	
		Recessive	GG + GT	166(97.1%)	163(99.4%)	1.00	0.095
			TT	5(2.9%)	1(0.6%)	0.2 (0.02–1.76)	
		Overdominant	GG + TT	139(81.3%)	147(89.6%)	1.00	0.029
			GT	32(18.7%)	17(10.4%)	0.5 (0.27–0.94)	
		Log-additive				0.47 (0.27–0.82)	0.005

## Discussion

End-stage renal disease is a complicated condition resulting from several genetic and environmental factors (Obrador et al., 2017). Abnormalities in lipid metabolism including high levels of triglyceride, elevation in isodose and high-dense, triglyceride-rich particles, and decreases in high-density lipoprotein cholesterol have been reported in patients with chronic renal disorder (Gromovsky et al., 2018). Genes that regulate lipid metabolism might be involved in the pathogenesis of ESRD. In the current study, we investigated the association between *FADS* polymorphisms and ESRD in a population of Iranian patients. *FADS* genotypes have been previously found to be associated with inflammation and coronary artery disorder (Martinelli et al., 2008). Chronic inflammation has been regarded as a risk factor frequently detected in patients with ESRD patients and might lead to atherosclerotic changes (Stenvinkel and Alvestrand, 2002). Therefore, *FADS* genes might provide the mechanical link between inflammation, ESRD, and coronary artery disorder. Moreover, polymorphisms in the *FADS* gene cluster have been demonstrated to modulate fatty acid levels and affect the pathogenesis of atopic disorders (Lattka et al., 2009).

Based on the results of the current study, the rs174556 of *FADS1* gene and rs99780 of *FADS2* gene were not associated with the risk of ESRD in any inheritance model. However, the rs7115739 of *FADS3* was associated with the risk of ESRD in all models except for the recessive model. T allele of this SNP was significantly less prevalent among cases compared with controls, therefore this allele can be regarded as a protective allele against ESRD. This SNP has been among variants identified in a scan of Inuit genomes for signatures of adaptation to an omega-3 polyunsaturated fatty acids-rich diet. Moreover, this SNP has been found to be associated with several metabolic and anthropometric features with a particularly large effect on weight and height (Fumagalli et al., 2015). Mechanistically, rs7115739 modulates fatty acid composition and might consequently influence the regulation of growth hormones (Fumagalli et al., 2015).

Consistently, GT and TT genotypes were found to decrease the risk of ESRD in the codominant model. In the dominant model, GT + TT status was associated with a lower risk of ESRD. Assessment of association between this SNP and risk of ESRD in the overdominant model revealed that the GT genotype decreases the risk of this condition. Therefore, the results of association analysis in different inheritance models consistently support the association between rs7115739 and the risk of ESRD. Although the underlying cause of the contribution of rs7115739 in the development of ESRD is not clear, the role of *FADS* genes in the modulation of inflammatory responses and the importance of these types of responses in the pathogenesis of ESRD suggest this mechanism as a possible way rs7115739 contributes in the pathogenesis of ESRD.

Taken together, the rs7115739 of *FADS3* is suggested as putative modulator of the risk of ESRD in the Iranian population. The results of this study should be confirmed in larger cohorts of patients. Moreover, functional studies are required to clarify the mechanistical points. Our study has some limitations regarding the lack of validation of results from an independent external population, lack of evaluation of close SNP in LD with associated polymorphisms, and cross-validation with random subsets of the data. Moreover, this is a pilot study where we tested only three SNP. Thus, we plan to conduct a follow up study where we will investigate more polymorphisms in the same region.

## Data Availability Statement

The original contributions presented in the study are included in the article/supplementary material, further inquiries can be directed to the corresponding authors.

## Ethics Statement

The studies involving human participants were reviewed and approved by the study protocol was approved by Ethical Committee of Shahid Beheshti University of Medical Sciences (IR. SBMU.UNRC.1398.10). The patients/participants provided their written informed consent to participate in this study.

## Author Contributions

MT and SG-F wrote the draft of the manuscript and revised the manuscript. AM performed the sample collection and clinical information. KH, MG, and AA performed the experiment. VK analyzed the data. All authors read and approved the submitted version.

## Conflict of Interest

The authors declare that the research was conducted in the absence of any commercial or financial relationships that could be construed as a potential conflict of interest.

## Publisher’s Note

All claims expressed in this article are solely those of the authors and do not necessarily represent those of their affiliated organizations, or those of the publisher, the editors and the reviewers. Any product that may be evaluated in this article, or claim that may be made by its manufacturer, is not guaranteed or endorsed by the publisher.

## Footnotes

1
https://www.ncbi.nlm.nih.gov/snp
